# Urelumab alone or in combination with rituximab in patients with relapsed or refractory B‐cell lymphoma

**DOI:** 10.1002/ajh.25757

**Published:** 2020-02-29

**Authors:** John Timmerman, Charles Herbaux, Vincent Ribrag, Andrew D. Zelenetz, Roch Houot, Sattva S. Neelapu, Theodore Logan, Izidore S. Lossos, Walter Urba, Gilles Salles, Radhakrishnan Ramchandren, Caron Jacobson, John Godwin, Cecilia Carpio, Deanne Lathers, Yali Liu, Jaclyn Neely, Satyendra Suryawanshi, Yoshinobu Koguchi, Ronald Levy

**Affiliations:** ^1^ UCLA Medical Center Los Angeles California; ^2^ Centre Hospitalier Régional Universitaire de Lille Lille France; ^3^ Institut Gustave Roussy Villejuif France; ^4^ Memorial Sloan Kettering Cancer Center New York New York; ^5^ CHU Rennes, Service Hématologie Clinique Rennes France; ^6^ INSERM Unité dʼInvestigation Clinique Rennes France; ^7^ The University of Texas MD Anderson Cancer Center Houston Texas; ^8^ Simon Cancer Center Indiana University Indianapolis Indiana; ^9^ University of Miami Miller School of Medicine Sylvester Comprehensive Cancer Center Miami Florida; ^10^ Earle A. Chiles Research Institute Providence Cancer Center Portland Oregon; ^11^ Hospices Civils de Lyon Université de Lyon Lyon France; ^12^ Karmanos Cancer Institute Detroit Michigan; ^13^ Dana‐Farber Cancer Institute Harvard Medical School Boston Massachusetts; ^14^ Hospital Universitari Vall dʼHebron Universitat Autònoma de Barcelona Barcelona Spain; ^15^ Bristol‐Myers Squibb Princeton New Jersey; ^16^ Stanford University School of Medicine Stanford California

## Abstract

Urelumab, a fully human, non‐ligand binding, CD137 agonist IgG4 monoclonal antibody, enhances T‐cell and natural killer‐cell antitumor activity in preclinical models, and may enhance cytotoxic activity of rituximab. Here we report results in patients with relapsed or refractory diffuse large B‐cell lymphoma (DLBCL), follicular lymphoma (FL), and other B‐cell lymphomas, in phase 1 studies evaluating urelumab alone (NCT01471210) or combined with rituximab (NCT01775631). Sixty patients received urelumab (0.3 mg/kg IV Q3W, 8 mg IV Q3W, or 8 mg IV Q6W); 46 received urelumab (0.1 mg/kg, 0.3 mg/kg, or 8 mg IV Q3W) plus rituximab 375 mg/m^2^ IV QW. The maximum tolerated dose (MTD) of urelumab was determined to be 0.1 mg/kg or 8 mg Q3W after a single event of potential drug‐induced liver injury occurred with urelumab 0.3 mg/kg. Treatment‐related AEs were reported in 52% (urelumab: grade 3/4, 15%) and 72% (urelumab + rituximab: grade 3/4, 28%); three led to discontinuation (grade 3 increased AST, grade 4 acute hepatitis [urelumab]; one death from sepsis syndrome [urelumab plus rituximab]). Objective response rates/disease control rates were 6%/19% (DLBCL, n = 31), 12%/35% (FL, n = 17), and 17%/42% (other B‐cell lymphomas, n = 12) with urelumab and 10%/24% (DLBCL, n = 29) and 35%/71% (FL, n = 17) with urelumab plus rituximab. Durable remissions in heavily pretreated patients were achieved; however, many were observed at doses exceeding the MTD. These data show that urelumab alone or in combination with rituximab demonstrated manageable safety in B‐cell lymphoma, but the combination did not enhance clinical activity relative to rituximab alone or other current standard of care.

## 1 INTRODUCTION

Diffuse large B‐cell lymphoma (DLBCL) and follicular lymphoma (FL) comprise approximately half of all cases of non‐Hodgkin lymphoma (NHL).^1^ DLBCL, the most common type of NHL (≈30% of cases), is a heterogeneous, aggressive lymphoma,^1^, ^2^ whereas FL is an indolent lymphoma accounting for approximately 22% of NHL cases.^1^ The introduction of chemoimmunotherapy, including high‐dose chemotherapy regimens in combination with the CD20‐directed monoclonal antibody rituximab, has improved outcomes in patients with DLBCL and FL.^1^, ^3^, ^4^ The majority of patients with DLBCL can be cured with first‐line therapy; however, approximately one‐third of all patients are refractory to treatment or relapse afterward.^2^, ^3^ In contrast, most patients with FL experience disease progression (PD) after treatment, with recurrent relapses characterized by shorter remissions with each successive line of therapy.^5^ Patients with FL who progress within 24 months of diagnosis after first‐line chemoimmunotherapy have significantly shorter overall survival.^6^ Patients with relapsed FL may also become refractory to chemoimmunotherapy or undergo histological transformation to a more aggressive NHL subtype.^5^, ^7^ Prognosis remains poor for patients with relapsed or refractory (R/R) DLBCL or FL^2^, ^5^, ^6^; therefore, novel, more effective regimens are needed for these R/R populations. Therapeutic blockade of checkpoint pathway inhibitory receptors has demonstrated efficacy in multiple malignancies, including in patients with R/R classic Hodgkin lymphoma.^8^, ^9^ However, an unmet need remains in patients with R/R B‐cell lymphomas, as variable clinical benefit has been observed with single‐agent checkpoint pathway blockade.^10^, ^11^, ^12^

Additional immunotherapy approaches targeting immunoregulatory receptors, including agonist antibodies against costimulatory molecules such as CD137 (4‐1BB), may enhance antitumor immunity in patients with cancer.^13^, ^14^, ^15^ Signaling via CD137, a costimulatory member of the tumor necrosis factor receptor (TNFR) superfamily, can lead to induction of cytokines, protection from activation‐induced cell death, and upregulation of cytotoxic T‐cell activity and may also reduce the infiltration of regulatory T cells into tumors.^14^, ^15^, ^16^, ^17^, ^18^, ^19^ In murine lymphoma models, agonist anti‐CD137 treatment led to long‐lasting antitumor activity mediated by natural killer and CD8 T cells.^19^

Urelumab is a fully human, non‐ligand binding, CD137 agonist immunoglobulin‐γ 4 (IgG4) monoclonal antibody, that was evaluated as monotherapy or in combination with other immunotherapies or targeted agents in multiple phase 1/2 clinical trials.^16^, ^20^, ^21^, ^22^ In an integrated safety analysis of three urelumab monotherapy studies (NCT00309023, NCT00612664, and NCT01471210), urelumab doses ≥1 mg/kg every 3 weeks (Q3W) were shown to be associated with more frequent transaminitis.^16^ Therefore, in these and all subsequent studies of urelumab, a lower dose range (<1 mg/kg Q3W) was evaluated, and liver toxicities were closely monitored. Results from urelumab monotherapy and combination studies suggested limited clinical activity in patients with advanced solid tumors; however, preliminary activity was observed in patients with lymphoma.^20^, ^21^ Here, we report final results in patients with R/R DLBCL, FL, and other types of B‐cell NHL treated in two phase 1 studies evaluating urelumab alone or in combination with rituximab (NCT01471210; NCT01775631).

## 2 METHODS

### 2.1 Study design and treatments

NCT01471210 (CA186‐011) was an open‐label, phase 1 study evaluating the safety, pharmacokinetics (PK), immunoregulatory activity, and antitumor activity of urelumab in patients with advanced and/or metastatic solid tumors and R/R B‐cell NHL across 22 active sites in France, Germany, Spain, and the United States. The results from expansion cohorts in patients with R/R B‐cell NHL are reported here. Patients with DLBCL, FL, or other types of B‐cell NHL were treated with urelumab 0.3 mg/kg intravenously (IV) Q3W for ≤8 doses or urelumab 8 mg IV Q3W (≤8 doses; equivalent to 0.1 mg/kg in an 80‐kg patient) or Q6W (≤4 doses; Figure S1).

NCT01775631 (CA186‐017) was an open‐label, phase 1b study evaluating the safety, PK, pharmacodynamics, and antitumor activity of urelumab in combination with rituximab in patients with R/R B‐cell NHL across 12 active sites in the United States. Patients with DLBCL or FL were treated with urelumab 0.3 mg/kg, 0.1 mg/kg, or 8 mg IV Q3W (≤8 doses) + rituximab 375 mg/m^2^ IV QW (≤8 doses; administered during the first 4 weeks of each 12‐week cycle) during escalation and urelumab 8 mg IV Q3W (≤8 doses) plus rituximab 375 mg/m^2^ IV QW (≤8 doses; administered during the first 4 weeks of each 12‐week cycle) during expansion (Figure S1).

In both studies, patients were treated until PD, unacceptable toxicity, or withdrawal of consent. Treatment beyond PD, defined by the International Working Group (IWG) Response Criteria for NHL,^23^ was permitted in patients experiencing clinical benefit without signs of clinical deterioration or intolerance of therapy per investigator discretion. Patients completing approximately 24 weeks of treatment and entering follow‐up for reasons other than treatment‐related toxicity with ongoing disease control, and subsequent confirmed PD within 12 months of the last dose, were eligible for retreatment for an additional 24 weeks.

The study protocols were approved by the institutional review board or independent ethics committee of each participating institution. The studies were conducted in accordance with the Declaration of Helsinki and Good Clinical Practice as defined by the International Conference on Harmonization. All patients provided written informed consent prior to enrollment.

### 2.2 Patients

In CA186‐011, eligible patients with B‐cell NHL had R/R disease after ≥1 prior line of standard therapy per IWG Response Criteria for NHL.^23^ In CA186‐017, eligible patients had CD20^+^ B‐cell NHL with measurable disease per IWG Response Criteria for NHL^23^ that was refractory to or had relapsed after ≥1 prior line of standard therapy. Patients in the expansion phase must have received ≥1 prior multiagent chemotherapy regimen and must have had R/R disease after prior rituximab alone or in combination with chemotherapy. In both studies, patients had to be aged ≥18 years with an ECOG performance status of 0 or 1 and were required to provide pre‐ and on‐treatment biopsies or fine‐needle aspirates.

Patients with central nervous system lymphoma, active autoimmune disease (or a documented history of autoimmune disease or a syndrome that required systemic steroids or immunosuppressive medications), known or suspected HIV or hepatitis (or history of hepatitis), evidence of active infection, or history of clinically significant cardiac disease were not eligible for enrollment in either study.

Anticancer therapy, nononcology live viral vaccine therapy (for the prevention of infectious diseases), surgery (unless minor [ie, biopsies]), radiotherapy, or the use of immunosuppressive medications or immunosuppressive doses of systemic corticosteroids (doses >10 mg/day of prednisone or equivalent) or growth‐factor treatments were not permitted within 28 days of dosing in either study. Prior treatment with agents targeting immune checkpoints (eg, programmed death [PD]‐1, programmed death ligand 1 [PD‐L1], PD‐L2, lymphocyte‐activation gene 3, or cytotoxic T lymphocyte antigen‐4) was permitted after a washout period of >100 days from the last dose in a subset of patients treated in CA186‐011. And, it was permitted only during the early portion of CA186‐017 after a washout period of >28 days; in the final CA186‐017 protocol, prior treatment with checkpoint pathway inhibitors was prohibited. Prior treatment with agents targeting T‐cell costimulatory pathways (eg, CD137, glucocorticoid‐induced TNFR‐related protein, OX40) was not permitted in either study.

### 2.3 Endpoints and assessments

The primary endpoint was safety and tolerability of urelumab (CA186‐011) and urelumab plus rituximab (CA186‐017). Secondary endpoints included PK, immunogenicity, and antitumor activity. Key exploratory endpoints included pharmacodynamic analyses.

Adverse events (AEs) were assessed during treatment, for ≥60 days after the last dose of urelumab, and for ≥110 days after the last dose of rituximab according to the National Cancer Institute Common Terminology Criteria for Adverse Events version 4.0. Determination of the maximum tolerated dose (MTD) in CA186‐011 was based on the incidence of drug‐related dose‐limiting toxicities (DLTs) during the first 9 weeks of therapy. It was defined as the highest dose at which <33% of patients experienced a DLT and <20% of patients experienced a hepatic nonhematologic DLT. This was with no event of Hyʼs law (any drug‐related alanine aminotransferase [ALT] or aspartate aminotransferase [AST] > 3 × upper limit of normal [ULN] accompanied by concurrent total bilirubin >2 × ULN without cholestasis) occurring at any time. In CA186‐017, the MTD of urelumab plus rituximab was based on the incidence of DLTs during the first 43 days of therapy and was defined as the highest dose at which <33% of patients experienced a DLT, with no event of Hyʼs law occurring at any time.

Pharmacokinetics and immunogenicity summary statistics were determined in serum samples collected at baseline, at protocol‐defined time points throughout treatment, at the end of treatment, and during the follow‐up period (30‐ and 60‐day visits only for urelumab; 30‐, 60‐, and 120‐day visits for rituximab).

Tumor response was evaluated by the investigator per IWG Response Criteria for NHL^23^ at baseline, during treatment (every 9 weeks [CA186‐011] or every 12 weeks [CA186‐017]) until PD or treatment discontinuation using computed tomography, magnetic resonance imaging, or fluorodeoxyglucose positron emission tomography (PET). In patients with bone marrow involvement at baseline, a bone marrow biopsy or aspirate was required to confirm a complete response.

Changes in immune‐related gene expression in whole blood (supplemental methods) and serum levels of immune factors during treatment were evaluated by quantitative polymerase chain reaction and quantitative multiplexed immunoassays (Myriad RBM, Austin, TX), respectively, in samples collected at baseline and at protocol‐specified time points throughout cycles 1 and 2.

### 2.4 Statistical analyses

Descriptive statistics were used to characterize baseline demographics, safety, PK, immunogenicity, and pharmacodynamics. Clopper‐Pearson 95% two‐sided confidence intervals were used to estimate objective response rate (ORR; defined as best response of complete remission or partial remission) and disease control rate (DCR; defined as best response of complete remission, partial remission, or stable disease). Kaplan‐Meier methodology was used to estimate duration of response (DOR; defined as the time from the first response to PD or death. Duration of response was censored at the last tumor assessment if the patient had not progressed or died at the time of the analysis), progression‐free survival (PFS; defined as the time from the first dose to PD or death from any cause), and overall survival (OS; defined as the time from the first dose to death from any cause).

All patients who received ≥1 dose of urelumab (CA186‐011) or urelumab plus rituximab (CA186‐017) were included in baseline demographic, safety, and antitumor analyses. For PK, immunogenicity, and pharmacodynamic analyses, only patients with adequate baseline and postbaseline PK, immunogenicity, or pharmacodynamic data were included.

## 3 RESULTS

### 3.1 Patient population and disposition

A total of 106 patients with R/R B‐cell NHL were treated with urelumab (n = 60: DLBCL, n = 31; FL, n = 17; other B‐cell lymphomas, n = 12) or urelumab plus rituximab (n = 46: DLBCL, n = 29; FL, n = 17) (Table 1). Median age ranged from 52 to 76 years across dose cohorts in both studies. Patients were mostly male (59%) and predominantly white (93%). Most patients with DLBCL had received ≥3 prior systemic therapies (urelumab monotherapy, 52%; urelumab plus rituximab, 69%); fewer patients with FL (urelumab monotherapy, 29%; urelumab plus rituximab, 53%) and other types of B‐cell NHL (urelumab monotherapy, 42%) had received ≥3 prior systemic therapies (Table 1).

**Table 1:** Baseline demographics and prior therapy in patients treated with urelumab or urelumab plus rituximab

	DLBCL								FL								Other B‐cell lymphomas			
	Urelumab				Urelumab Plus Rituximab				Urelumab				Urelumab Plus Rituximab				Urelumab			
	8 mg Q3W (n = 10)	8 mg Q6W (n = 18)	0.3 mg/kg (n = 3)	Total (N = 31)	0.1 mg/kg (n = 14)	8 mg Q3W (n = 12)	0.3 mg/kg (n = 3)	Total (N = 29)	8 mg Q3W (n = 10)	8 mg Q6W (n = 2)	0.3 mg/kg (n = 5)	Total (N = 17)	0.1 mg/kg (n = 3)	8 mg Q3W (n = 11)	0.3 mg/kg (n = 3)	Total (N = 17)	8 mg Q3W (n = 5)	8 mg Q6W (n = 5)	0.3 mg/kg (n = 2)	Total (N = 12)
Age, median (range), y	71.5 (31‐80)	70 (51‐85)	62 (60‐72)	70 (31‐85)	64 (42‐78)	53 (22‐75)	76 (68‐87)	63 (22‐87)	69 (45‐81)	73.5 (70‐77)	58 (37‐64)	66 (37‐81)	70 (67‐87)	65 (48‐75)	70 (64‐70)	67 (48‐87)	68 (59‐84)	73 (57‐80)	52 (50‐54)	68 (50‐84)
<65 y, n (%)	4 (40)	4 (22)	2 (67)	10 (32)	7 (50)	9 (75)	0	16 (55)	3 (30)	0	5 (100)	8 (47)	0	5 (45)	1 (33)	6 (35)	2 (40)	1 (20)	2 (100)	5 (42)
Male, n (%)	6 (60)	11 (61)	1 (33)	18 (58)	7 (50)	9 (75)	1 (33)	17 (59)	8 (80)	1 (50)	2 (40)	11 (65)	1 (33)	7 (64)	1 (33)	9 (53)	3 (60)	3 (60)	2 (100)	8 (67)
Race, n (%)																				
White	10 (100)	18 (100)	3 (100)	31 (100)	13 (93)	9 (75)	3 (100)	25 (86)	10 (100)	2 (100)	5 (100)	17 (100)	2 (67)	9 (82)	3 (100)	14 (82)	5 (100)	5 (100)	2 (100)	12 (100)
Other/not reported	0	0	0	0	1 (7)	3 (25)	0	4 (14)	0	0	0	0	1 (33)	2 (18)	0	3 (18)	0	0	0	0
Prior therapy, n (%)																				
Radiotherapy	5 (50)	3 (17)	2 (67)	10 (32)	4 (29)	6 (50)	1 (33)	11 (38)	2 (20)	1 (50)	2 (40)	5 (29)	1 (33)	6 (55)	1 (33)	8 (47)	0	1 (20)	0	1 (8)
Systemic therapy	10 (100)	18 (100)	3 (100)	31 (100)	14 (100)	12 (100)	3 (100)	29 (100)	10 (100)	2 (100)	5 (100)	17 (100)	3 (100)	11 (100)	3 (100)	17 (100)	5 (100)	5 (100)	2 (100)	12 (100)
Prior lines of systemic therapy, n (%)																				
1	3 (30)	8 (44)	1 (33)	12 (39)	0	0	0	0	2 (20)	0	4 (80)	6 (35)	1 (33)	2 (18)	0	3 (18)	1 (20)	0	1 (50)	2 (17)
2	1 (10)	2 (11)	0	3 (10)	3 (21)	5 (42)	1 (33)	9 (31)	3 (30)	2 (100)	1 (20)	6 (35)	0	5 (45)	0	5 (29)	2 (40)	3 (60)	0	5 (42)
≥3	6 (60)	8 (44)	2 (67)	16 (52)	11 (79)	7 (58)	2 (67)	20 (69)	5 (50)	0	0	5 (29)	2 (67)	4 (36)	3 (100)	9 (53)	2 (40)	2 (40)	1 (50)	5 (42)

Abbreviations: DLBCL, diffuse large B‐cell lymphoma; PD‐1, programmed death 1 protein; Q3W, every 3 weeks; Q6W, every 6 weeks.

At the time of the final database locks (CA0186‐011, June 3, 2016; CA0186‐017, October 4, 2016), 101 patients had discontinued treatment with urelumab (n = 57; 95%) or urelumab plus rituximab (n = 44; 96%), primarily due to PD (urelumab, 75%; urelumab plus rituximab, 63%; Table S1). Median durations of treatment were 9.4 weeks (range, 3.0‐97.3 weeks) with urelumab and 9.2 weeks (range, 3.0‐67.0 weeks) with urelumab plus rituximab (Table S2).

### 3.2 Safety

The MTD was determined to be urelumab 0.1 mg/kg or 8 mg Q3W after a single event of potential drug‐induced liver injury (pDILI) occurred in a patient treated with urelumab 0.3 mg/kg in study CA186‐011. Following this event, enrollment into the 0.3 mg/kg dose level was halted, and all subsequent patients enrolled into the CA186‐011 and CA186‐017 studies were treated with lower doses. In CA186‐011, dosing was reduced to 0.1 mg/kg for all ongoing patients and to 8 mg Q3W or Q6W for all subsequent patients. In CA186‐017, dosing was reduced to 0.1 mg/kg or 8 mg Q3W.

With urelumab monotherapy, treatment‐related AEs (TRAEs) were reported in 52% of patients (grade 3/4, 15%) (Table 2), with the most frequent TRAEs (≥10%, any grade) being fatigue (any grade, 15%; no grade 3/4 events) and neutropenia (any grade, 12%; grade 3/4, 12%). A higher frequency of any‐grade TRAEs was observed with urelumab 0.3 mg/kg (any grade, 80%; grade 3/4, 10%) compared with urelumab 8 mg Q3W (any grade, 48%; grade 3/4, 24%) and urelumab 8 mg Q6W (any grade, 44%; grade 3/4, 8%). The TRAEs leading to discontinuation of urelumab monotherapy included grade 3 increased AST (n = 1) and grade 4 acute hepatitis (n = 1). No treatment‐related deaths were reported.

**Table 2:** Treatment‐related adverse event summary in patients treated with urelumab or urelumab plus rituximab

	Urelumab								Urelumab Plus Rituximab							
	8 mg Q3W (n = 25)		8 mg Q6W (n = 25)		0.3 mg/kg (n = 10)		All patients (N = 60)		0.1 mg/kg (n = 17)		8 mg Q3W (n = 23)		0.3 mg/kg (n = 6)		All patients (N = 46)	
	Any grade	Grade 3/4	Any grade	Grade 3/4	Any grade	Grade 3/4	Any grade	Grade 3/4	Any grade	Grade 3/4	Any grade	Grade 3/4	Any grade	Grade 3/4	Any grade	Grade 3/4
All TRAEs, n (%)	12 (48)	6 (24)	11 (44)	2 (8)	8 (80)	1 (10)	31 (52)	9 (15)	13 (76)	7 (41)	16 (70)	5 (22)	4 (67)^a^	1 (17)	33 (72)^a^	13 (28)
TRAEs (> 2 patients in either study), n (%)																
Hematologic AEs																
Neutropenia	5 (20)	5 (20)	2 (8)	2 (8)	0	0	7 (12)	7 (12)	5 (29)	5 (29)	0	0	1 (17)	0	6 (13)	5 (11)
Thrombocytopenia	2 (8)	1 (4)	1 (4)	0	0	0	3 (5)	1 (2)	2 (12)	0	1 (4)	1 (4)	2 (33)	1 (17)	5 (11)	2 (4)
Anemia	1 (4)	0	0	0	0	0	1 (2)	0	1 (6)	0	1 (4)	1 (4)	1 (17)	0	3 (7)	1 (2)
Leukopenia	0	0	0	0	0	0	0	0	3 (18)	2 (12)	0	0	1 (17)	1 (17)	4 (9)	3 (7)
Lymphopenia	0	0	0	0	0	0	0	0	1 (6)	1 (6)	1 (4)	0	1 (17)	1 (17)	3 (7)	2 (4)
Nonhematologic AEs																
Fatigue	1 (4)	0	4 (16)	0	4 (40)	0	9 (15)	0	3 (18)	0	6 (26)	1 (4)	0	0	9 (20)	1 (2)
AST increased	1 (4)	1 (4)	1 (4)	0	1 (10)	0	3 (5)	1 (2)	2 (12)	0	4 (17)	0	1 (17)	0	7 (15)	0
Increased blood alkaline phosphatase	1 (4)	0	0	0	2 (20)	0	3 (5)	0	0	0	0	0	0	0	0	0
Nausea	2 (8)	0	0	0	0	0	2 (3)	0	1 (6)	0	3 (13)	0	1 (17)	0	5 (11)	0
Diarrhea	2 (8)	0	0	0	0	0	2 (3)	0	1 (6)	0	3 (13)	0	0	0	4 (9)	0
ALT increased	1 (4)	0	0	0	0	0	1 (2)	0	1 (6)	0	4 (17)	1 (4)	1 (17)	0	6 (13)	1 (2)
Infusion‐related reaction	0	0	1 (4)	0	0	0	1 (2)	0	1 (6)	0	2 (9)	0	1 (17)	0	4 (9)	0
Vomiting	0	0	0	0	0	0	0	0	1 (6)	1 (6)	2 (9)	0	0	0	3 (7)	1 (2)
Constipation	0	0	0	0	0	0	0	0	1 (6)	0	2 (9)	0	0	0	3 (7)	0
Lipase increased	0	0	0	0	0	0	0	0	1 (6)	0	1 (4)	0	1 (17)	0	3 (7)	0
Serious TRAEs, n (%)	2 (8)	2 (8)	1 (4)	0	1 (10)	1 (10)	4 (7)	3 (5)	0	0	1 (4)	1 (4)	1 (17)^a^	0	2 (4)^a^	1 (2)
TRAEs leading to discontinuation, n (%)	1 (4)^b^	1 (4)^b^	0	0	1 (10)^c^	1 (10)^c^	2 (3)^b^^,^ c	2 (3)^b^^,^ c	0	0	0	0	1 (17)^a^	0	1 (2)^a^	0

Abbreviations: AE, adverse event; AST, aspartate aminotransferase; DLBCL, diffuse large B‐cell lymphoma; FL, follicular lymphoma; Q3W, every 3 weeks; Q6W, every 6 weeks; TRAE, treatment‐related adverse event.

a Death due to sepsis syndrome in a patient with FL who had received treatment with nivolumab 32 days prior to study entry.

b Grade 3 increased AST in patient with DLBCL.

c Grade 4 acute hepatitis in patient with FL.

With urelumab plus rituximab, 72% of patients experienced a TRAE (grade 3/4, 28%; Table 2), with the most frequently reported events (≥10% of any grade) being fatigue (any grade, 20%; grade 3/4, 2%), increased AST (any grade, 15%; no grade 3/4 events), increased ALT (any grade, 13%; grade 3/4, 2%), neutropenia (any grade, 13%; grade 3/4, 11%), thrombocytopenia (any grade, 11%; grade 3/4, 4%), and nausea (any grade, 11%; no grade 3/4 events). One patient treated with urelumab 0.3 mg/kg plus rituximab died from treatment‐related sepsis syndrome/cytokine release syndrome. This event occurred in a patient with bulky, rituximab‐refractory FL who had initiated treatment with urelumab 0.3 mg/kg plus rituximab 32 days after progressing on nivolumab. Lymphadenopathy began to regress rapidly in this patient prior to death. Laboratory findings included grade 4 transaminase elevations, grade 1 hyperbilirubinemia, and elevations in serum cytokines including interleukin (IL)‐6, IL‐10, TNFα, and monocyte chemoattractant protein‐1; a postmortem examination revealed moderate hepatitis and lymph nodes showing necrotic tumor largely replaced by CD3^+^ T cells, fibrosis, and macrophages.

### 3.3 Pharmacokinetics

Urelumab PK parameters increased proportionately with dose and were not altered substantially when urelumab was combined with rituximab (Table S3). Following a 1‐hour IV infusion, maximum concentrations of urelumab were reached at a median time of 1.17 to 2.00 hours with urelumab monotherapy and 2.33 to 3.00 hours with urelumab plus rituximab. The geometric mean maximum concentration (C_max_) of urelumab was similar in the 0.1 mg/kg and 8 mg flat‐dose treatment groups in both studies (C_max_, 2.065 μg/mL with urelumab 0.1 mg/kg compared with 2.78 and 2.18 μg/mL with urelumab 8 mg Q3W, and 2.12 μg/mL with urelumab 8 mg Q6W) and increased approximately 3‐fold in the 0.3 mg/kg treatment groups (C_max_, 6.025 and 8.15 μg/mL).

### 3.4 Immunogenicity

Sixteen percent of patients treated with urelumab 8 mg Q3W monotherapy, 16% of patients treated with urelumab 8 mg Q6W monotherapy, and 30% of patients treated with urelumab 0.3 mg/kg monotherapy were antidrug antibody (ADA) positive after treatment (Table S4). Overall, ADA positivity did not appear to affect urelumab safety. No patients were ADA positive after treatment with urelumab plus rituximab.

### 3.5 Efficacy

With urelumab monotherapy, ORR and DCR were respectively 6% and 19% in patients with DLBCL (n = 31), 12% and 35% in patients with FL (n = 17), and 17% and 42% in patients with other types of B‐cell NHL (n = 12; Table 3). Half of the responses occurred in patients treated with urelumab 0.3 mg/kg, which exceeded what was subsequently determined to be the MTD. Median DOR was not reached in patients with DLBCL or FL; however, in patients with other types of B‐cell NHL, median DOR was 18.1 weeks (Table 3). In the 31 patients with DLBCL treated with urelumab monotherapy, median PFS was 8.1 weeks, and median OS was 45.6 weeks (Table 4). In the 17 patients with FL treated with urelumab monotherapy, median PFS was 8.9 weeks, and median OS was not reached. In patients with other types of B‐cell NHL (n = 12), median PFS was 13.4 weeks, and median OS was not reached.

**Table 3:** Response and disease control (by investigator) in patients treated with urelumab or urelumab plus rituximab

	DLBCL^1^								FL^1^								Other B‐cell lymphomas^1^			
	Urelumab				Urelumab Plus Rituximab				Urelumab				Urelumab Plus Rituximab				Urelumab			
	8 mg Q3W (n = 10)	8 mg Q6W (n = 18)	0.3 mg/kg (n = 3)	Total (N = 31)	0.1 mg/kg (n = 14)	8 mg Q3W (n = 12)	0.3 mg/kg (n = 3)	Total (N = 29)	8 mg Q3W (n = 10)	8 mg Q6W (n = 2)	0.3 mg/kg (n = 5)	Total (N = 17)	0.1 mg/kg (n = 3)	8 mg Q3W (n = 11)	0.3 mg/kg (n = 3)	Total (N = 17)	8 mg Q3W (n = 5)	8 mg Q6W (n = 5)	0.3 mg/kg (n = 2)	Total (N = 12)
Best overall response, n (%)																				
Complete remission	0	0	0	0	1 (7)	1 (8)	0	2 (7)	0	0	1 (20)	1 (6)	0	1 (9)	1 (33)	2 (12)	1 (20)	1 (20)	0	2 (17)
Partial remission	0	1 (6)	1 (33)	2 (6)	1 (7)	0	0	1 (3)	0	0	1 (20)	1 (6)	3 (100)	1 (9)	0	4 (24)	0	0	0	0
Stable disease	2 (20)	1 (6)	1 (33)	4 (13)	1 (7)	2 (17)	1 (33)	4 (14)	2 (20)	1 (50)	1 (20)	4 (24)	0	5 (45)	1 (33)	6 (35)	0	1 (20)	2 (100)	3 (25)
Relapse/progressive disease	5 (50)	13 (72)	1 (33)	19 (61)	9 (64)	8 (67)	2 (67)	19 (66)	8 (80)	1 (50)	2 (40)	11 (65)	0	3 (27)	0	3 (18)	3 (60)	2 (40)	0	5 (42)
Death prior to assessment	‐	‐	‐	‐	0	1 (8)	0	1 (3)	‐	‐	‐	‐	0	0	1 (33)	1 (6)	‐	‐	‐	‐
Other	‐	‐	‐	‐	2 (14)	0	0	2 (7)	‐	‐	‐	‐	0	0	0	0	‐	‐	‐	‐
Unable to determine	3 (30)	3 (17)	0	6 (19)	0	0	0	0	0	0	0	0	0	1 (9)	0	1 (6)	1 (20)	1 (20)	0	2 (17)
Confirmed ORR, n (%) 95% CI	0 0‐30.8	1 (6) 0.1‐27.3	1 (33) 0.8‐90.6	2 (6) 0.8‐21.4	2 (14) 1.8‐42.8	1 (8) 0.2‐38.5	0 0‐70.8	3 (10) 2.2‐27.4	0 0‐30.8	0 0‐84.2	2 (40) 5.3‐85.3	2 (12) 1.5‐36.4	3 (100) 29.2‐100	2 (18) 2.3‐51.8	1 (33) 0.8‐90.6	6 (35) 14.2‐61.7	1 (20) 0.5‐71.6	1 (20) 0.5‐71.6	0 0‐84.2	2 (17) 2.1‐48.4
Confirmed DCR, n (%) 95% CI	2 (20) 2.5‐55.6	2 (11) 1.4‐34.7	2 (67) 9.4‐99.2	6 (19) 7.5‐37.5	3 (21) 4.7‐50.8	3 (25) 5.5‐57.2	1 (33) 0.8‐90.6	7 (24) 10.3‐43.5	2 (20) 2.5‐55.6	1 (50) 1.3‐98.7	3 (60) 14.7‐94.7	6 (35) 14.2‐61.7	3 (100) 29.2‐100	7 (64) 30.8‐89.1	2 (67) 9.4‐99.2	12 (71) 44.0‐89.7	1 (20) 0.5‐71.6	2 (40) 5.3‐85.3	2 (100) 15.8‐100.0	5 (42) 15.2‐72.3
Median DOR, weeks Range	NR	NR 29.3+ to 29.3+	21.1 21.1‐21.1	NR 21.1‐29.3+	NR 2.3‐51.1+	NR 51.3+ to 51.3+	‐	NR 2.3‐51.3+	NR	NR	NR 0.1+ to 25.0+	NR 0.1+ to 25.0+	18.1 15.0‐74.0+	NR 0.1+ to 30.3+	NR 92.3+ to 92.3+	NR 0.1+ to 92.3+	18.1 18.1‐18.1	NR 14.4+ to 14.4+	NR	18.1 14.4‐18.1
Median TTR, weeks Range	NR 27.4+ to 72.4+	NR 9.3‐72.3+	NR 9.4‐103.9+	NR 9.3‐103.9+	NR 12.0‐124.7+	NR 12.0‐92.3+	‐	NR 12.0‐124.7+	NR 28.0+ to 67.3+	NR 52.3+ to 53.4+	NR 7.3‐108.7+	NR 7.3‐108.7+	13.1 12.3‐25.6	NR 12.1‐85.6+	25.4 16.6+ to 25.4	NR 12.1‐85.6+	NR 8.3‐55.3+	NR 13.3‐44.4+	NR 88.9+ to 93.7+	NR 8.3‐93.7+

Abbreviations: +, ongoing response; DCR, disease control rate; DLBCL, diffuse large B‐cell lymphoma; DOR, duration of response; FL, follicular lymphoma; IWG, International Working Group; NHL, non‐Hodgkin lymphoma; NR, not reached; ORR, objective response rate; Q3W, every 3 weeks; Q6W, every 6 weeks; TTR, time to response.

Response was evaluated per IWG Response Criteria in NHL in all efficacy‐evaluable patients.

**Table 4:** Progression‐free survival and overall survival in patients treated with urelumab or urelumab plus rituximab

	DLBCL								FL								Other B‐cell lymphomas			
	Urelumab				Urelumab Plus Rituximab				Urelumab				Urelumab Plus Rituximab				Urelumab			
	8 mg Q3W (n = 10)	8 mg Q6W (n = 18)	0.3 mg/kg (n = 3)	Total (N = 31)	0.1 mg/kg (n = 14)	8 mg Q3W (n = 12)	0.3 mg/kg (n = 3)	Total (N = 29)	8 mg Q3W (n = 10)	8 mg Q6W (n = 2)	0.3 mg/kg (n = 5)	Total (N = 17)	0.1 mg/kg (n = 3)	8 mg Q3W (n = 11)	0.3 mg/kg (n = 3)	Total (N = 17)	8 mg Q3W (n = 5)	8 mg Q6W (n = 5)	0.3 mg/kg (n = 2)	Total (N = 12)
PFS																				
Events, n (%)	8 (80)	13 (72)	3 (100)	24 (77)	13 (93)	11 (92)	3 (100)	27 (93)	10 (100)	1 (50)	2 (40)	13 (76)	2 (67)	4 (36)	1 (33)	7 (41)	5 (100)	3 (60)	1 (50)	9 (75)
Median (95% CI), weeks	8.1 (2.9‐17.0)	8.0 (3.1‐8.4)	24.6 (9.1‐30.4)	8.1 (5.3‐9.1)	11.6 (4.4‐12.3)	7.4 (3.0‐12.3)	12.4 (6.6‐25.3)	9.0 (5.6‐12.3)	8.2 (5.1‐9.3)	NR (8.3 to −)	NR (7.0 to −)	8.9 (6.4‐15.0)	40.4 (30.3 to −)	35.1 (2.3 to −)	NR (3.7 to −)	40.4 (12.1 to −)	8.4 (1.7‐26.3)	15.4 (4.1 to −)	NR (22.4 to −)	13.4 (2.4‐26.3)
OS																				
Events, n (%)	4 (40)	7 (39)	1 (33)	12 (39)	7 (50)	8 (67)	1 (33)	16 (55)	2 (20)	0	0	2 (12)	0	1 (9)	1 (33)	2 (12)	2 (40)	1 (20)	0	3 (25)
Median (95% CI), weeks	31.1 (2.9 to −)	30.7 (21.7 to −)	NR (45.6 to −)	45.6 (28.9 to −)	45.7 (15.6‐98.0)	19.6 (10.0 to −)	NR (13.3 to −)	23.9 (18.1‐98.0)	64.3 (28.7‐64.3)	−	−	NR (28.7 to −)	−	NR (10.6 to −)	NR (3.7 to −)	NR	42.6 (2.4 to −)	NR (19.6 to −)	−	NR (19.6 to −)

Abbreviations: DLBCL, diffuse large B‐cell lymphoma; FL, follicular lymphoma; NR, not reached; OS, overall survival; PFS, progression‐free survival.

With urelumab plus rituximab, ORR and DCR were respectively 10% and 24% in patients with DLBCL (n = 29) and 35% and 71% in patients with FL (n = 17; Table 3); responses were observed across all doses. Median DOR was not reached in patients with DLBCL or FL treated with the combination (Table 3). In the 29 patients with DLBCL treated with urelumab plus rituximab, median PFS was 9.0 weeks, and median OS was 23.9 weeks (Table 4). In the 17 patients with FL treated with the combination, median PFS was 40.4 weeks, and median OS was not reached.

Several patients achieved durable partial or complete remissions or stable disease with urelumab as monotherapy or in combination with rituximab. One patient with DLBCL treated with urelumab 8 mg Q6W maintained a partial remission with a PFS of approximately 18 months prior to death at age 84 due to metastatic prostate cancer as second malignancy. Another patient with DLBCL treated with urelumab 8 mg Q3W plus rituximab was alive at the last follow‐up (July 2019), with stable disease for >55 months without subsequent treatment; since the end of study, this patientʼs liver lesion has become smaller, and his PET scan was negative. A patient with FL who had progressed after multiple rounds of rituximab‐based therapy (with the last progression following a 14‐month response with bendamustine) achieved complete remission with urelumab 0.3 mg/kg plus rituximab; this response was still ongoing at 56 months at the last follow‐up (June 2019). Additionally, the patient with FL who experienced grade 4 chemical hepatitis after receiving several doses of urelumab 0.3 mg/kg (the DLT that defined the MTD) had a partial remission of a large thoracic mass that lasted for 13 months.

### 3.6 Pharmacodynamics

Expression of interferon‐γ (IFN‐γ)‐induced genes, including C‐X‐C motif chemokine ligand 9 (*CXCL9*; also known as monokine induced by IFN‐γ) and guanylate binding protein 1 (*GBP1*), increased after treatment with urelumab monotherapy and urelumab plus rituximab (Figure S2A, B). Although samples were limited, a trend was observed towards greater induction of *CXCL9* and *GBP1* with urelumab 0.3 mg/kg monotherapy 1 week after the first and/or second dose and greater induction of *GBP1* with urelumab 0.3 mg/kg plus rituximab after cycle one, day 5. Mean expression levels returned to baseline in samples collected prior to administration of the second dose. Many cytokines, including CXCL10 (also known as IFN‐γ‐induced protein 10), were also transiently induced with urelumab monotherapy or urelumab plus rituximab (Figure S2C). However, due to a limited number of samples available at baseline for urelumab 0.3 mg/kg, dose dependency could not be determined. Overall, no association was observed between peripheral IFN‐γ‐induced changes and response; however, correlative analyses were limited by a small sample size.

## 4 DISCUSSION

The rationale for evaluation of urelumab in hematologic malignancies was based on preclinical analyses of human primary lymphomas, including DLBCL and FL.^19^ Bulk tumor samples from patients with lymphoma showed overexpression of CD137 mRNA compared with nonlymphoma samples and were infiltrated with CD137^+^ T cells, while tumor B cells were uniformly negative for CD137.^19^ The population of CD137^+^ tumor‐infiltrating T cells may be a source of tumor‐reactive cells that could be stimulated by CD137 agonism.^19^ Moreover, in murine lymphoma models, anti‐CD137 treatment led to durable antitumor activity as monotherapy.^19^

In the CA186‐011 and CA186‐017 phase 1 studies, the safety and antitumor activity of urelumab alone or in combination with rituximab were evaluated in patients with solid tumors (CA186‐011 only) and B‐cell NHL. These studies were designed to assess a lower urelumab dose range, with a focus on liver toxicities, due to liver injury and drug‐related deaths observed in prior studies that evaluated higher doses of urelumab.^16^ While the clinical mechanism is unclear, previously published preclinical data suggest that anti‐CD137‐induced liver toxicity may be partially due to infiltration of S100A4^+^ macrophages into the liver, following activation of CD8^+^ T cells and secretion of IFN‐γ.^24^, ^25^ The TRAEs leading to discontinuation included grade 3 increased AST in a patient treated with urelumab 8 mg Q3W, grade 4 acute hepatitis in a patient treated with urelumab 0.3 mg/kg, and one death from sepsis syndrome in a patient treated with urelumab 0.3 mg/kg plus rituximab. The sepsis syndrome was the only treatment‐related death reported in either study. Overall, the MTD was established as urelumab 0.1 mg/kg or 8 mg Q3W based on a pDILI reported in one patient treated with urelumab 0.3 mg/kg (CA186‐011). Despite this single event, liver toxicity was less frequent and severe in these studies than previously observed with higher urelumab doses.^16^

In the CA186‐017 study, urelumab in combination with rituximab did not enhance clinical activity relative to rituximab alone^26^, ^27^ or standard of care.^1^, ^3^ Although rituximab is generally evaluated in combination regimens in DLBCL, a previous study of rituximab monotherapy (eight doses) demonstrated an ORR of 37% in patients with DLBCL, including patients with R/R disease or those >60 years old without prior therapy;^27^ this ORR is higher than that observed with urelumab plus rituximab in patients with R/R DLBCL in this study (ORR, 10%). Moreover, in previous studies of rituximab monotherapy (four doses) in patients with R/R FL or low‐grade lymphoma, ORRs ranged from 36% to 48%,^26^ which are similar to or higher than that observed in patients with R/R FL treated with urelumab plus rituximab in this study (ORR, 35%). Cross‐study comparisons should be interpreted with caution because the patient populations are different, and the proportion of patients with prior rituximab treatment in studies CA186‐011 and CA186‐017 is likely higher than in the earlier studies noted above. Of note, the ORR observed in patients with FL treated with urelumab plus rituximab in this study was similar to that observed with another anti‐CD137 agonist, utomilumab, evaluated in combination with rituximab (four doses) in patients with rituximab‐refractory FL (ORR, 33% [n = 24, dose escalation] and 44% [n = 9, cohort expansion]).^28^ Variable trends of PFS and OS observed in CA186‐011 and CA186‐017 have also been reported in studies evaluating recommended regimens for R/R FL and DLBCL.^3^

In the CA186‐011 and CA186‐017 studies, antitumor activity, including several durable remissions, was observed with urelumab as monotherapy or in combination with rituximab. However, many of these responses were observed at doses that exceeded the MTD, suggesting that the limited clinical activity observed may be due to suboptimal CD137 agonism. Future studies evaluating next‐generation therapies that target CD137 with unique approaches to safely increase the dose/exposure of CD137 delivery are underway.^14^, ^29^ Specific approaches include but are not limited to bispecific antibodies engaging 4‐1BB and a tumor antigen/stromal component, intratumoral delivery, local nanoparticle‐anchored antibodies, and/or unique combination therapies.^14^, ^29^, ^30^, ^31^, ^32^ These strategies may lead to more efficacious CD137 therapy for patients with R/R B‐cell lymphoma, a population with a high unmet need.

## CONFLICT OF INTEREST

J. Timmerman: Advisory board member and/or consultant for Celgene, Kite/Gilead, Partner Therapeutics, and TeneoBio; research funding from Bristol‐Myers Squibb, Kite/Gilead, Merck, and Spectrum Pharmaceuticals.

C. Herbaux: Personal fees from AbbVie and Roche.

V. Ribrag: Advisory board member and/or consultant for MSD, Bristol‐Myers Squibb, Epizyme, Nanostring, Pharmamar, Servier, Gilead; research funding from ArgenX, AstraZeneca, Bristol‐Myers Squibb, Boehringer Ingelheim, Janssen Cilag, Merck, Novartis, Pfizer, Roche, and Sanofi; personal fees from Roche and Infinity; non‐financial support from AstraZeneca, Bayer, Bristol‐Myers Squibb, Boehringer Ingelheim, Johnson & Johnson, Lilly, MedImmune, Merck, NH TherAguix, Pfizer, and Roche.

R. Houot: Consultant for Bristol‐Myers Squibb, Novartis, Janssen, Celgene, and Kite/Gilead.

S. Neelapu: Advisory board member and/or consultant for Kite/Gilead, Merck, Celgene, Novartis, Unum Therapeutics, Pfizer, Precision BioSciences, Cell Medica, Allogene, Incyte, and Legend Biotech; research funding from Kite/Gilead, Merck, Bristol‐Myers Squibb, Cellectis, Poseida, Karus, Acerta, and Unum Therapeutics.

T. Logan: Advisory board member and/or consultant for Prometheus; research funding from Abbott, Abraxis, Acceleron, Amgen, Argos, AstraZeneca, Aveo, BioVex, Bristol‐Myers Squibb, Cerulean, Dynavax, Eisai, EMD Serono, GlaxoSmithKline, Iovance Biotherapeutics, Immatics, Lilly, MacroGenics, Millennium, MedImmune, Merck, Novartis, Peloton, Pfizer, Prometheus, Roche, Synta, Threshold, and Tracon.

I. Lossos: Advisory board member for Seattle Genetics and Janssen Scientific; personal fees from Verastem.

W. Urba: Advisory board member for AstraZeneca; research funding from Bristol‐Myers Squibb.

G. Salles: Advisory board member/symposia participant for Amgen, Bristol‐Myers Squibb, AbbVie, Janssen, Merck, Novartis, Gilead/Kite, Epizyme, Pfizer, Celgene, Roche, Takeda, Autolus, MorphoSys, Acerta, and Servier; consultant for Epizyme.

C. Jacobson: Consultant for Kite, Novartis, Celgene, Humanigen, Pfizer, and Precision BioSciences; research funding from Pfizer.

C. Carpio: Personal fees from Takeda; non‐financial support from Takeda, Celgene, and Gilead.

D. Lathers: Employee of Bristol‐Myers Squibb.

Y. Liu: Former employee of Bristol‐Myers Squibb.

J. Neely: Employee and stock owner of Bristol‐Myers Squibb.

S. Suryawanshi: Employee and stock owner of Bristol‐Myers Squibb.

Y. Koguchi: Research funding from Bristol‐Myers Squibb, Shimadzu Corporation, and Tesaro, a GlaxoSmithKline company.

R. Levy: Scientific advisory board for Five Prime, Corvus, Quadriga, BeiGene, GigaGen, TeneoBio, Sutro, Checkmate, Nurix, Dragonfly, Innate Pharma, Abpro, Apexigen, Nohla, Spotlight, Forty Seven Inc, xCella, Immunocore, and Walking Fish; research funding from Pfizer, Bristol‐Myers Squibb, and Pharmacyclics.

All other authors declare no conflict of interest.

## Supporting information


**Table S1** Patient disposition at end of treatment.
**Table S2**. Urelumab dose information summary.
**Table S3**. Pharmacokinetic summary in patients treated with urelumab or urelumab plus rituximab.
**Table S4**. Immunogenicity summary.
**Figure S1**. Study designs and objectives for evaluation of urelumab or urelumab plus rituximab in patients with lymphoma.
**Figure S2**. Peripheral changes in IFN‐γ‐induced genes and cytokines over time in patients treated with urelumab or urelumab plus rituximab.

## References

[1] NCCN Clinical Practice Guidelines in Oncology. B‐Cell Lymphomas. Version 1. 2019. National Comprehensive Cancer Network, Plymouth Meeting, PA. 10.6004/jnccn.2022.0015 35276674

[2] Roschewski M , Staudt LM , Wilson WH . Diffuse large B‐cell lymphoma‐treatment approaches in the molecular era. Nat Rev Clin Oncol. 2014;11(1):12‐23. 24217204 10.1038/nrclinonc.2013.197 PMC7709161

[3] Galaznik A , Huelin R , Stokes M , et al. Systematic review of therapy used in relapsed or refractory diffuse large B‐cell lymphoma and follicular lymphoma. Future Sci OA. 2018;4(7):FSO322. 30112190 10.4155/fsoa-2018-0049 PMC6088264

[4] Armitage JO , Gascoyne RD , Lunning MA , Cavalli F . Non‐Hodgkin lymphoma. Lancet. 2017;390(10091):298‐310. 28153383 10.1016/S0140-6736(16)32407-2

[5] Wudhikarn K , Link BK . Comparative effectiveness research in follicular lymphoma: current and future perspectives and challenges. J Comp Eff Res. 2014;3(1):95‐107. 24345259 10.2217/cer.13.86

[6] Casulo C , Byrtek M , Dawson KL , et al. Early relapse of follicular lymphoma after rituximab plus cyclophosphamide, doxorubicin, vincristine, and prednisone defines patients at high risk for death: an analysis from the National LymphoCare Study. J Clin Oncol. 2015;33(23):2516‐2522. 26124482 10.1200/JCO.2014.59.7534 PMC4879714

[7] Freedman A . Follicular lymphoma: 2018 update on diagnosis and management. Am J Hematol. 2018;93(2):296‐305. 29314206 10.1002/ajh.24937

[8] Opdivo (nivolumab) [package insert]. Princeton, NJ: Bristol‐Myers Squibb Company; 2019.

[9] Keytruda (pembrolizumab) [package insert]. Whitehouse Station, NJ: Merck Sharp & Dohme Corp; 2017.

[10] Ansell SM , Hurvitz SA , Koenig PA , et al. Phase I study of ipilimumab, an anti‐CTLA‐4 monoclonal antibody, in patients with relapsed and refractory B‐cell non‐Hodgkin lymphoma. Clin Cancer Res. 2009;15(20):6446‐6453. 19808874 10.1158/1078-0432.CCR-09-1339 PMC2763019

[11] Ansell SM , Minnema MC , Johnson P , et al. Nivolumab for relapsed/refractory diffuse large B‐cell lymphoma in patients ineligible for or having failed autologous transplantation: a single‐arm, phase II study. J Clin Oncol. 2019;37(6):481‐489. 30620669 10.1200/JCO.18.00766 PMC6528729

[12] Ding W , Laplant B , Witzig TE , et al. PD‐1 blockade with pembrolizumab in relapsed low grade non‐Hodgkin lymphoma. Blood. 2017;130:4055.

[13] Capece D , Verzella D , Fischietti M , Zazzeroni F , Alesse E . Targeting costimulatory molecules to improve antitumor immunity. J Biomed Biotechnol. 2012;2012:926321. 22500111 10.1155/2012/926321 PMC3303883

[14] Chester C , Sanmamed MF , Wang J , Melero I . Immunotherapy targeting 4‐1BB: mechanistic rationale, clinical results, and future strategies. Blood. 2018;131(1):49‐57. 29118009 10.1182/blood-2017-06-741041

[15] Yonezawa A , Dutt S , Chester C , Kim J , Kohrt HE . Boosting cancer immunotherapy with anti‐CD137 antibody therapy. Clin Cancer Res. 2015;21(14):3113‐3120. 25908780 10.1158/1078-0432.CCR-15-0263 PMC5422104

[16] Segal NH , Logan TF , Hodi FS , et al. Results from an integrated safety analysis of urelumab, an agonist anti‐CD137 monoclonal antibody. Clin Cancer Res. 2017;23(8):1929‐1936. 27756788 10.1158/1078-0432.CCR-16-1272

[17] Palazon A , Teijeira A , Martinez‐Forero I , et al. Agonist anti‐CD137 mAb act on tumor endothelial cells to enhance recruitment of activated T lymphocytes. Cancer Res. 2011;71(3):801‐811. 21266358 10.1158/0008-5472.CAN-10-1733

[18] Vinay DS , Kwon BS . 4‐1BB (CD137), an inducible costimulatory receptor, as a specific target for cancer therapy. BMB Rep. 2014;47(3):122‐129. 24499671 10.5483/BMBRep.2014.47.3.283 PMC4163883

[19] Houot R , Goldstein MJ , Kohrt HE , et al. Therapeutic effect of CD137 immunomodulation in lymphoma and its enhancement by Treg depletion. Blood. 2009;114(16):3431‐3438. 19641184 10.1182/blood-2009-05-223958 PMC2765679

[20] Segal NH , Sharma M , Le D , et al. Assessing the potential for enhanced antibody‐dependent cell‐mediated cytotoxicity by combining the CD137 antibody urelumab with rituximab or cetuximab in patients with refractory lymphoma or select advanced solid tumors. J Immunother Cancer. 2016;4(suppl 1):P267.

[21] Massarelli E , Segal NH , Ribrag V , et al. Clinical safety and efficacy assessment of the CD137 agonist urelumab alone and in combination with nivolumab in patients with hematologic and solid tumor malignancies. J Immunother Cancer. 2016;4(suppl 1):O7.

[22] Clinicaltrials.gov. https://www.clinicaltrials.gov/. Accessed February 24, 2020.

[23] Cheson BD , Pfistner B , Juweid ME , et al. Revised response criteria for malignant lymphoma. J Clin Oncol. 2007;25(5):579‐586. 17242396 10.1200/JCO.2006.09.2403

[24] Zhang J , Song K , Wang J , et al. S100A4 blockage alleviates agonistic anti‐CD137 antibody‐induced liver pathology without disruption of antitumor immunity. Oncoimmunology. 2018;7(4):e1296996. 29632708 10.1080/2162402X.2017.1296996 PMC5889198

[25] Bartkowiak T , Jaiswal AR , Ager CR , et al. Activation of 4‐1BB on liver myeloid cells triggers hepatitis via an interleukin‐27‐dependent pathway. Clin Cancer Res. 2018;24(5):1138‐1151. 29301830 10.1158/1078-0432.CCR-17-1847 PMC6752715

[26] Rituxan (rituximab) [package insert]. South San Francisco, CA: Biogen and Genentech USA, Inc; 2019.

[27] Coiffier B , Haioun C , Ketterer N , et al. Rituximab (anti‐CD20 monoclonal antibody) for the treatment of patients with relapsing or refractory aggressive lymphoma: a multicenter phase II study. Blood. 1998;92(6):1927‐1932. 9731049

[28] Gopal A , Levy R , Houot R , et al. A phase I study of utomilumab (PF‐05082566), a 4‐1BB/CD137 agonist, in combination with rituximab in patients with CD20+ non‐Hodgkinʼs lymphoma. Hematol Oncol. 2017;35(suppl 2):267.

[29] Chu DT , Bac ND , Nguyen KH , et al. An update on anti‐CD137 antibodies in immunotherapies for cancer. Int J Mol Sci. 2019;20(8):1822. 10.3390/ijms20081822 PMC6515339 31013788

[30] Compte M , Harwood SL , Munoz IG , et al. A tumor‐targeted trimeric 4‐1BB‐agonistic antibody induces potent anti‐tumor immunity without systemic toxicity. Nat Commun. 2018;9(1):4809. 30442944 10.1038/s41467-018-07195-w PMC6237851

[31] Qi X , Li F , Wu Y , et al. Optimization of 4‐1BB antibody for cancer immunotherapy by balancing agonistic strength with FcgammaR affinity. Nat Commun. 2019;10(1):2141. 31105267 10.1038/s41467-019-10088-1 PMC6526162

[32] Claus C , Ferrara C , Xu W , et al. Tumor‐targeted 4‐1BB agonists for combination with T cell bispecific antibodies as off‐the‐shelf therapy. Sci Transl Med. 2019;11(496):pii: eaav5989. 10.1126/scitranslmed.aav5989 PMC7181714 31189721

