# Supplementary material for: Urelumab alone or in combination with rituximab in patients with relapsed or refractory B‐cell lymphoma
Source: Am J Hematol. 2020 Feb 29;95(5):510–20. doi: 10.1002/ajh.25757 (PMC7383599; doi:10.1002/ajh.25757)
Supplement: Supplementary file 1 — Table S1 Patient disposition at end of treatment. Table S2. Urelumab dose information summary. Table S3. Pharmacokinetic summary in patients treated with urelumab or urelumab plus rituximab. Table S4. Immunogenicity summary. Figure S1. Study designs and objectives for evaluation of urelumab or urelumab plus rituximab in patients with lymphoma. Figure S2. Peripheral changes in IFN‐γ‐induced genes and cytokines over time in patients treated with urelumab or urelumab plus rituximab. [file AJH-95-510-s001.docx]

# Supporting Information

**Supplemental Methods**

Immune-related genes evaluated by quantitative polymerase chain reaction included MX dynamin-like GTPase 1 (*MX1*), 2'-5'-oligoadenylate synthetase 2 (*OAS2*), radical SAM domain-containing 2 (*RSAD2*), chemokine (*C-X-C motif*) ligand 9 (*CXCL9*), and guanylate-binding protein 1 (*GBP1*). RNA levels were measured in whole blood; peripheral blood mononuclear cells and lymphocytes were not isolated prior to RNA extraction. Target gene expression levels were normalized to the housekeeping gene glyceraldehyde-3-phosphate dehydrogenase (*GAPDH*).

**Supplemental Tables**

**Table S1. Patient disposition at end of treatment**

|  | **Urelumab** | | | | **Urelumab Plus Rituximab** | | | |
| --- | --- | --- | --- | --- | --- | --- | --- | --- |
|  | **8 mg Q3W**  **(n = 25)** | **8 mg Q6W**  **(n = 25)** | **0.3 mg/kg  (n = 10)** | **All Patients**  **(N = 60)** | **0.1 mg/kg**  **(n = 17)** | **8 mg Q3W**  **(n = 23)** | **0.3 mg/kg**  **(n = 6)** | **All Patients**  **(N = 46)** |
| **Continuing treatment, n (%)** | 1 (4) | 2 (8) | 0 | 3 (5) | 0 | 2 (9) | 0 | 2 (4) |
| **Not continuing treatment, n (%)** | 24 (96) | 23 (92) | 10 (100) | 57 (95) | 17 (100) | 21 (91) | 6 (100) | 44 (96) |
| **Reason for treatment discontinuation, n (%)** |  |  |  |  |  |  |  |  |
| **Disease progression** | 22 (88) | 18 (72) | 5 (50) | 45 (75) | 14 (82) | 12 (52) | 3 (50) | 29 (63) |
| **Study drug toxicity** | 1 (4) | 0 | 1 (10) | 2 (3) | 0 | 1 (4) | 1 (17) | 2 (4) |
| **AE unrelated to study drug** | 0 | 1 (4) | 1 (10) | 2 (3) | 1 (6) | 0 | 0 | 1 (2) |
| **Maximum clinical benefit** | 0 | 0 | 0 | 0 | 0 | 1 (4) | 1 (17) | 2 (4) |
| **Patient decision** | 0 | 1 (4) | 0 | 1 (2) | 1 (6) | 1 (4) | 0 | 2 (4) |
| **Patient withdrew consent** | 0 | 2 (8) | 0 | 2 (3) | 0 | 1 (4) | 0 | 1 (2) |
| **Patient no longer met criteria** | 0 | 0 | 0 | 0 | 0 | 1 (4) | 0 | 1 (2) |
| **Other** | 1 (4) | 1 (4) | 3 (30) | 5 (8) | 1 (6) | 4 (17) | 1 (17) | 6 (13) |

AE, adverse event; Q3W, every 3 weeks; Q6W, every 6 weeks.

**Table S2. Urelumab dose information summary**

|  | **Urelumab** | | | | **Urelumab Plus Rituximab** | | | |
| --- | --- | --- | --- | --- | --- | --- | --- | --- |
|  | **8 mg Q3W**  **(n = 25)** | **8 mg Q6W**  **(n = 25)** | **0.3 mg/kg  (n = 10)** | **All Patients**  **(N = 60)** | **0.1 mg/kg**  **(n = 17)** | **8 mg Q3W**  **(n = 23)** | **0.3 mg/kg**  **(n = 6)** | **All Patients**  **(N = 46)** |
| **Median number of doses (range)** | 3 (1-8) | 2 (1-8) | 7.5 (2-31) | 2 (1-31) | 4 (1-12) | 3 (1-20) | 2 (1-4) | 3 (1-20) |
| **Median duration of therapy (range), weeks** | 9.0 (3.0-25.0) | 12.0 (6.0-51.0) | 23.2 (6.3-97.3) | 9.4 (3.0-97.3) | 11.9 (3.0-41.0) | 9.0 (3.0-67.0) | 5.9 (3.0-12.3) | 9.2 (3.0-67.0) |
| **Relative dose intensity, n (%)** |  |  |  |  |  |  |  |  |
| **< 50%** | 0 | 0 | 2 (20) | 2 (3) | 0 | 0 | 0 | 0 |
| **≥ 50% to < 70%** | 0 | 0 | 3 (30) | 3 (5) | 0 | 0 | 0 | 0 |
| **≥ 70% to < 90%** | 3 (12) | 0 | 0 | 3 (5) | 5 (29) | 1 (4) | 0 | 6 (13) |
| **≥ 90% to < 110%** | 22 (88) | 25 (100) | 5 (50) | 52 (87) | 11 (65) | 22 (96) | 6 (100) | 39 (85) |
| **≥ 110%** | 0 | 0 | 0 | 0 | 1 (6) | 0 | 0 | 1 (2) |

Q3W, every 3 weeks; Q6W, every 6 weeks.

**Table S3. Pharmacokinetic summary in patients treated with urelumab or urelumab plus rituximab**

|  | **Urelumab** | | | **Urelumab Plus Rituximab** | | |
| --- | --- | --- | --- | --- | --- | --- |
|  | **8 mg Q3W** | **8 mg Q6W** | **0.3 mg/kg** | **0.1 mg/kg** | **8 mg Q3W** | **0.3 mg/kg** |
| **C_max_, mean, µg/mL [n]**  **(%CV)** | 2.18 [19]  (35) | 2.12 [15]  (25) | 6.03 [10]  (15) | 2.07 [17]  (40) | 2.78 [21]  (34) | 8.15 [6]  (18) |
| **C_tau_, mean, µg/mL [n]**  **(%CV)** | 0.096 [10] (45) | 0.050 [7]  (65) | 0.109 [3]  (104) | 0.062 [15]  (39) | 0.104 [14]  (27) | 0.308 [5]  (49) |
| **AUC (𝜏), mean, µg • h/mL [n]**  **(%CV)** | 234 [13]  (36) | 271 [13]  (39) | 455 [3]  (59) | 197 [17]  (30) | 249 [19]  (41) | 908 [6]  (35) |
| **T_max_, median, hr [n]**  **(min-max)** | 1.17 [19] (0.92-24) | 1.32 [15]  (0.58-25) | 2.00 [10] (1.0-4.0) | 2.33 [17]  (1.0-9.0) | 2.00 [21]  (1.0-5.0) | 3.00 [6]  (2.0-4.0) |

AUC, area under the curve; C_max_, maximum concentration; C_tau_, Ctrough; %CV, percent coefficient of variation; max, maximum; min, minimum; Q3W, every 3 weeks; Q6W, every 6 weeks; 𝜏, dosing interval; T_max_, time to maximum concentration.

**Table S4. Immunogenicity summary**

|  | **Urelumab** | | | | **Urelumab Plus Rituximab** | | | |
| --- | --- | --- | --- | --- | --- | --- | --- | --- |
|  | **8 mg Q3W**  **(n = 25)** | **8 mg Q6W**  **(n = 25)** | **0.3 mg/kg  (n = 10)** | **All Patients^a^**  **(N = 123)** | **0.1 mg/kg**  **(n = 17)** | **8 mg Q3W**  **(n = 23)** | **0.3 mg/kg**  **(n = 6)** | **All Patients**  **(N = 46)** |
| **Baseline ADA positive, n (%)** | 0 | 0 | 0 | 3 (2) | 0 | 1 (4) | 0 | 1 (2) |
| **ADA positive, n (%)** | 4 (16) | 4 (16) | 3 (30) | 41 (33) | 0 | 0 | 0 | 0 |
| **Persistent** | 1 (4) | 2 (8) | 3 (30) | 16 (13) | 0 | 0 | 0 | 0 |
| **Only at last sample** | 3 (12) | 1 (4) | 0 | 23 (19) | 0 | 0 | 0 | 0 |
| **Other** | 0 | 1 (4) | 0 | 2 (2) | 0 | 0 | 0 | 0 |
| **With neutralizing** | 0 | 1 (4) | 1 (10) | 9 (7) | 0 | 0 | 0 | 0 |
| **ADA negative, n (%)** | 18 (72) | 14 (56) | 6 (60) | 69 (56) | 15 (88) | 15 (65) | 5 (83) | 35 (76) |

ADA, antidrug antibody; Q3W, every 3 weeks; Q6W, every 6 weeks.

^a^Includes all patients (with solid tumor and lymphoma) treated with urelumab with available ADA assessment at baseline and postbaseline.

**Supplemental Figure**

**Figure S1. Study designs and objectives for evaluation of urelumab or urelumab plus rituximab in patients with lymphoma**

**
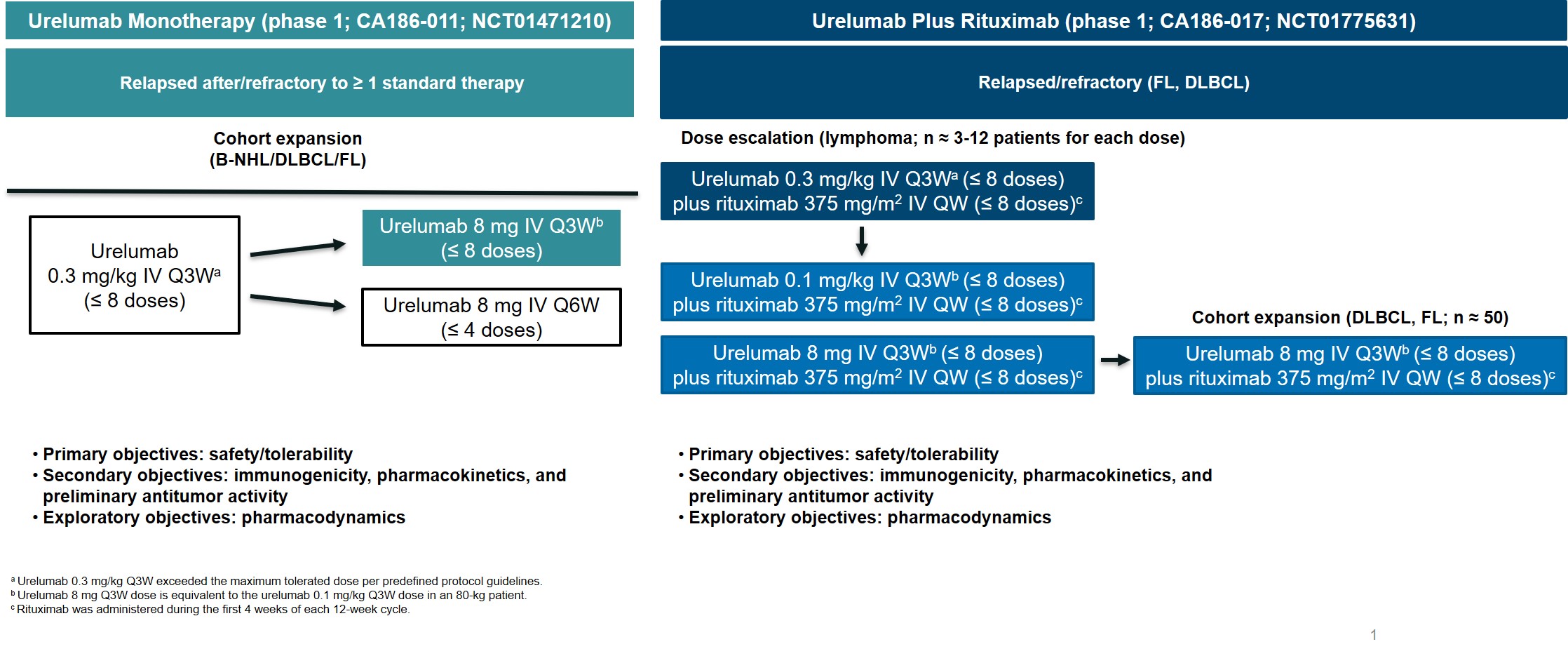
**

**Figure S2. Peripheral changes in IFN-γ-induced genes and cytokines over time in patients treated with urelumab or urelumab plus rituximab**

**
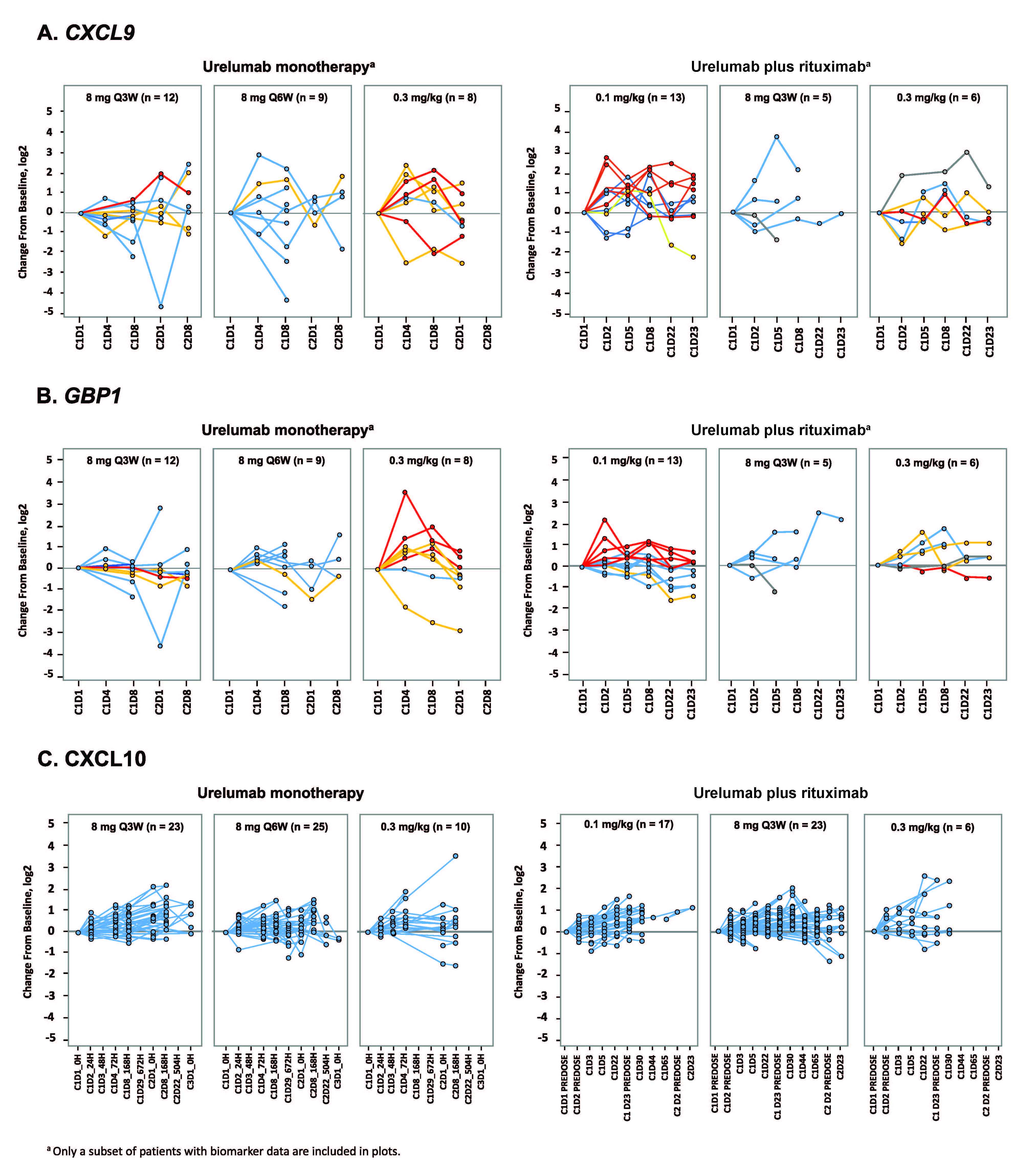
**

**Supplemental Figure Legend**

**Figure S1. Study designs and objectives for evaluation of urelumab or urelumab plus rituximab in patients with lymphoma**. Expansion cohorts from the open-label phase 1 CA186-011 study (NCT01471210) in patients with relapsed or refractory B-cell NHL (DLBCL, FL, other types) were treated with urelumab 0.3 mg/kg IV Q3W for ≤ 8 doses or urelumab 8 mg IV Q3W (≤ 8 doses) or Q6W (≤ 4 doses). Dose escalation and expansion cohorts from the open-label phase 1b CA186-017 study (NCT01775631) in patients with relapsed or refractory B-cell NHL (DLBCL or FL) were treated with urelumab 0.3 mg/kg, 0.1 mg/kg, or 8 mg IV Q3W (≤ 8 doses) plus rituximab 375 mg/m^2^ IV QW (≤ 8 doses; administered during the first 4 weeks of each 12-week cycle) during escalation and urelumab 8 mg IV Q3W (≤ 8 doses) plus rituximab 375 mg/m^2^ IV QW (≤ 8 doses; administered during the first 4 weeks of each 12-week cycle) during expansion. Patients completing approximately 24 weeks of treatment and entering follow-up for reasons other than treatment-related toxicity with ongoing disease control and subsequent confirmed PD within 12 months of the last dose were eligible for retreatment for an additional 24 weeks. Key study objectives are shown. B-NHL, other B-cell non-Hodgkin lymphomas; DLBCL, diffuse large B-cell lymphoma; FL, follicular lymphoma; IV, intravenous; NHL, non-Hodgkin lymphoma; QW, once weekly; Q3W, every 3 weeks; Q6W, every 6 weeks.

**Figure S2. Peripheral changes in IFN-ɣ–induced genes and cytokines over time in patients treated with urelumab or urelumab plus rituximab**

Plots show change from baseline (log 2) in expression of the IFN-ɣ–induced genes, *CXCL9* (panel A) and *GBP1* (panel B), in whole blood and CXCL10 (panel C) in serum by urelumab dose. In panels A and B, target gene expression levels were normalized to the housekeeping gene glyceraldehyde-3-phosphate dehydrogenase. Best overall response is depicted by color: red, complete or partial remission; yellow, stable disease; blue, relapse or progressive disease; black, death prior to disease assessment. IFN, interferon; Q3W, every 3 weeks; Q6W, every 6 weeks.
